# Ovarian clear cell carcinoma: open questions on the management and treatment algorithm

**DOI:** 10.1093/oncolo/oyae325

**Published:** 2025-01-23

**Authors:** Roberta Rosso, Margherita Turinetto, Fulvio Borella, Nicolas Chopin, Pierre Meeus, Alexandra Lainè, Isabelle Ray-Coquard, Olivia Le Saux, Domenico Ferraioli

**Affiliations:** Department of Gynecology and Obstetrics, Azienda Sanitaria Ospedaliera Santa Croce e Carle, Cuneo, Italy; Department of Oncology, University of Turin, Turin, Italy; Gynecology and Obstetrics Unit 1U, Department of Surgical Science, AOU Città della Salute e della Scienza di Torino, Sant’Anna Hospital, Turin, Italy; Léon Bérard Cancer Center, Department of Surgical and Medical Oncology-Lyon, Université Claude Bernard Lyon 1, Lyon, France; Léon Bérard Cancer Center, Department of Surgical and Medical Oncology-Lyon, Université Claude Bernard Lyon 1, Lyon, France; Léon Bérard Cancer Center, Department of Surgical and Medical Oncology-Lyon, Université Claude Bernard Lyon 1, Lyon, France; Léon Bérard Cancer Center, Department of Surgical and Medical Oncology-Lyon, Université Claude Bernard Lyon 1, Lyon, France; Léon Bérard Cancer Center, Department of Surgical and Medical Oncology-Lyon, Université Claude Bernard Lyon 1, Lyon, France; Léon Bérard Cancer Center, Department of Surgical and Medical Oncology-Lyon, Université Claude Bernard Lyon 1, Lyon, France

**Keywords:** ovarian cancer, clear cell, clear cell ovarian carcinoma, management, treatment, target therapy, prognosis

## Abstract

Ovarian clear cell carcinoma (OCCC) accounts for ~10% of all epithelial ovarian cancers and is considered a different entity from the more common high-grade serous ovarian carcinoma (HGSC), with distinct clinical presentations, different risk, and prognostic factors, and specific molecular features. Most OCCCs are diagnosed at an early stage and show favorable outcomes, in contrast to those diagnosed at advanced stages, which exhibit intrinsic resistance to platinum-based chemotherapy regimens and a very poor prognosis. The standard treatment of advanced OCCC is currently based on primary debulking surgery followed by platinum-based chemotherapy according to recent international guidelines. However, these recommendations are extrapolated from several trials mainly featuring a large cohort of HGSC, with only a small minority of OCCC. Because of its rarity, many questions remain unanswered regarding the surgical and medical treatment. Lymph node staging, fertility-sparing treatment, the use of targeted therapies and radiotherapy as well as the adjuvant treatment for early-stage disease and second or further lines of chemotherapy are still under debate. This review aims to address these unresolved issues, by providing a comprehensive overview of the current data on this disease, and to suggest possible directions for future research.

**Implications for Practice**

Ovarian clear cell carcinoma (OCCC) is a very rare subtype of epithelial ovarian cancer (EOC). Recommendations for OCCC treatment are extrapolated from several trials mainly featuring a large cohort of high-grade serous EOC or from retrospective studies. Because of its rarity and the difficulty of designing high-quality longitudinal studies, the best management of OCCC is still under debate. We present a review aimed at addressing these unresolved issues, providing a comprehensive overview of the current data on this disease, and suggesting possible directions for further studies.

## Introduction

Ovarian cancer (OC) is the eigth most common malignancy in women, with 313 959 new cases (1.6% of all cancers) and 207 252 deaths (2.1% of all cancers) in 2020.^1^ Ovarian clear cell carcinoma (OCCC) is a subtype of epithelial OC (EOC) that accounts for ~10% of all cases. Most OCCCs are diagnosed at early stages and show a favorable outcome, in contrast to those diagnosed in advanced stages, which have intrinsic resistance to platinum-based chemotherapy and a poor prognosis.^2^ OCCC has a higher incidence in Asia (10%-30%) than in Western countries (5%-12%).^3-5^ This ethnic difference may be explained by environmental factors, genetic mutations, or specific biological pathways, but the exact causes are unclear.^6,7^ OCCC has different clinical presentation, prognostic factors, and molecular characteristics compared with high-grade serous ovarian carcinoma (HGSC).^8,9^

Endometriosis, diagnosed in about half of OCCC patients, is associated with a 3-fold increased risk of developing OCCC.^10-13^ The endometriosis-related OC risk is related to inflammation, IL-6 production, and inactivation of tumor suppressor genes such as *ARID1A* and *PTEN* or activation of oncogenic KRAS and PI3K pathways.^14-17^ Hereditary conditions represent one of the main risk factors for developing EOC, accounting for about 23% of cases.^7^ While *BRCA1* and *BRCA2* genes germline mutations are associated with EOC, with a prevalence of 28.5% in HGSC and only 2.1% in OCCC, Lynch syndrome is more frequent in endometrioid or clear cell subtypes, accounting for ~25% and 12% of cases, respectively.^18,19^

The management of OCCC poses several challenges and controversies; therefore, there is an urgent need to develop alternative and personalized treatment strategies.

This review aims to provide a comprehensive overview of the current data on the management of OCCC and suggest future research directions.

## Methods

A comprehensive systematic literature search was performed; the electronic databases PubMed and Web of Science were searched, with a timeline up to December 2023, without time limitations.

The search strategy included the use of Mesh terms and keywords related to the subject, including “ovarian clear cell” OR “OCCC”; “cancer” OR “tumor” OR “neoplasm” OR “carcinoma” OR “malignancy”; and “surgery” OR “radiotherapy” OR “chemotherapy” OR “target therapy.” The search was limited to human studies published in full-length articles. Only papers published in the English language were considered. To supplement our search, we manually reviewed the citations of relevant original articles and review articles.

## CA125

Cancer antigen 125 (CA125) is elevated in ~75% of patients with HGSC; however, its diagnostic role is less significant in OCCC, where it is elevated in only about 57% of cases.^20^ Several studies have shown for OCCC a correlation between higher levels of CA125 and advanced disease, suboptimal debulking surgery, and platinum resistance, as well as worse progression-free survival (PFS) and overall survival (OS).^21-23^ Patients who achieved postoperative normalization of CA125 levels had significantly better survival rates compared with those with decreased but still elevated CA125 values (5-year PFS rates: 57.9% vs 45.2%, *P* =.046; 5-year OS rates: 81.4% vs 54.4%, *P* =.016) or those with increased CA125 values (5-year PFS rates: 57.9% vs 29.2%, *P* =.001; 5-year OS rates, 81.4% vs 33.6%, *P* <.001). These findings define postoperative CA125 levels as an independent risk factor for disease-free survival (DFS) and OS.^24^ The diagnostic role of CA19.9 value is debated; however, its value remains an independent risk factor for survival in patients with normal postoperative Ca125 levels.^24^

As traditional markers are not highly accurate in diagnosing OCCC, 2 new potential serum biomarkers have been proposed: TFPI2 and A2160. TFPI2 appears to have the ability to differentiate OCCC from other ovarian diseases, including other EOC subtypes, benign tumors, and endometriomas, with a sensitivity and specificity of 71.4% and 91.9%, respectively.^25^ A more recent analysis has confirmed these results, demonstrating the significance of TFPI2 in distinguishing OCCC from other EOC with a specificity of 79.5% (95% CI, 73.3-84.8), which is notably superior to that of Ca125, which stands at 24.9% (95% CI, 19.1-31.4).^26^ Another study assessed the role of A2160 to differentiate early-stage OCCC from endometriomas, reporting a sensitivity of 87% and a specificity of 94%. TFPI2 and A2160 may have potential significance in the early diagnosis of OCCC.^27^

## Prognosis

OCCC patients are mostly diagnosed at early stages rather than advanced stages (45%-81% at stage I vs 4%-35% at stage III).^28,29^ The recent literature shows different prognoses for early and advanced OCCC (5-year PFS and OS: 89% and 90% for stage I vs 22% and 46% for stage III, respectively).^30,31^ It is suggested that patients with early-stage OCCC have a similar or better prognosis compared with those with HSOC (5-year OS: 86.4% vs 85.3% for stage I).^8^ The relapse risk was the lowest for IA/IB/IC1 CC compared with HSOC, but OCCC had poor sensitivity to chemotherapy after relapse.^32,33^ FIGO stage I OCCC patients had a 3-year OS of 90.1% (93.5% for stage IA and 85.9% for stage IC).^2^ Interestingly, analyzing the FIGO stage IC subpopulation, the 3-year OS rates varied significantly: 96.2% for those with capsule rupture only and 71.9% for those with surface involvement. For stage IB, the 5-year disease-specific survival decreased to 56.3%. This survival rate is significantly lower than the one observed in other histological subtypes, suggesting that stage IB OCCC patients may have a worse prognosis compared with other histological subtypes.^8^

A recent review comparing outcomes of OCCC and HSOC has confirmed these results in early-stage disease and it has underlined the worse 5-year OS rates of advanced stages OCCC compared with HSOC (31.5% vs 35.0% stage III).^34^ The poorest prognosis of OCCC compared with HSOC has also been confirmed in relapsed settings with a 5-year survival rate of 13.2% versus 18.2%, respectively.^35,36^

Several studies have demonstrated the positive impact of complete debulking surgery on survival outcomes (HR = 17.2; 95% CI, 6.9-43.5) and the poorer prognosis of patients with residual disease.^37,38^

Lymphovascular space involvement may be an independent predictor of disease recurrence and death, particularly in endometriosis-associated OCCC.^39^

OCCC associated with endometriosis is diagnosed in younger patients, with less lymph node involvement, lower Ca125 levels, and platinum sensitivity.^11,31,40^ Some studies reported better OS and PFS rates in these patients and considered endometriosis a favorable prognostic factor.^11^ However, recent studies did not confirm endometriosis as an independent prognostic factor in OCCC.^39,41,42^ Conversely, other authors associated endometriosis with a worse prognosis.^43^

In recent years, the role of systemic inflammatory response (SIR) markers in OCCC has also been explored. These markers encompass the neutrophil-to-lymphocyte ratio (NLR), monocyte-to-lymphocyte ratio (MLR), and platelet-to-lymphocyte ratio, and they seem to be related to prognosis.^44^ In particular, high NLR and low MLR are associated with advanced-stage disease, presence of metastases, platinum resistance, and poor prognosis.^45,46^ The prognostic role of SIR markers has also been suggested in 2 studies investigating early-stage EOCs that included clear cell histotypes.^47,48^

The prognostic relevance of biological and molecular features in OCCC is still being investigated. Patients with mismatch repair gene deficiency (MMRd) might experience a better prognosis due to tumor immunogenicity, while patients with low ARID1A expression or aberrant p53 expression have worse outcomes.^49-51^

Some authors have investigated the molecular characterization of stage I EOCs. They found that stage I OCCC had a specific expression of some miRNA (miR-30a-3p and miR-30a-5p) involved in cell cycle regulation. Stage I OCCC shows specific expression differences for genes involved in extracellular structure organization, metabolic, and catabolic processes compared with other histotypes.^52,53^ Moreover, stage I OCCC is characterized by high activity of JUNB, E2F2, and FOS.^54^ Another study investigated the genome distribution of somatic copy number alterations (SCNAs) in stage I EOC and found that some recurrent SCNAs were more frequent in OCCC and were related to a worse prognosis.^55^

## Therapeutic strategies

### Surgical approaches

Primary debulking surgery followed by platinum-based chemotherapy represents the standard of treatment for OCCC, while neoadjuvant chemotherapy (NACT) followed by interval debulking surgery (IDS) is an alternative for those patients who are not suitable for upfront surgery. Surgical staging includes total hysterectomy with bilateral salpingo-oophorectomy, peritoneal biopsies, infragastric or infracolic omentectomy, bilateral pelvic and para-aortic lymphadenectomy, and peritoneal washing.^56^

In advanced disease, any surgical procedures such as bowel resection and/or appendectomy, stripping of the diaphragm or other peritoneal surfaces, splenectomy, partial cystectomy and/or ureteroneocystostomy, partial hepatectomy, partial gastrectomy, cholecystectomy, and/or distal pancreatectomy should be considered to achieve complete cytoreduction.^56,57^ Debulking surgery resulting in no macroscopic residual disease remains the main prognostic factor.^38,39^

Three multicentric randomized clinical trials evaluated the role of NACT followed by IDS for advanced EOC; however, OCCC was underrepresented (1.5%-6%).^58-60^ Therefore, it is controversial to apply the results of these trials to OCCC.^37,61^

## Lymph node dissection in early OCCC

Systematic pelvic and para-aortic lymph node dissection (LND) plays a key role in staging OCCC, but its prognostic value remains unclear.^4^ Different authors reported no association between pelvic and para-aortic LND and improved survival in pT1-pT2 OCCC patients.^62-64^ On the contrary, Yamazaki et al. identified a correlation between systematic LND and better DFS in 127 pT1-pT2 OCCC patients.^65^ MITO-9 study demonstrated that LND was associated with improved OS in advanced stages but not in early stages.^66^ A retrospective series suggested that the excision of more than 10 lymph nodes might correlate with improved prognosis, although the data did not reach statistical significance.^67^ Takei et al. reported that the removal of ≥35 lymph nodes was an independent predictive factor for improved DFS in stage I OCCC.^68^

Furthermore, the lymph nodal ratio (LNR; positive lymph nodes out of total removed lymph nodes) was also studied as a prognostic factor. Nie et al. evaluated the significance of LNR in stage III OCCC patients undergoing primary debulking surgery with systematic LND. In this cohort, the 5-year PFS rate was 32.4% for patients with LNR ≤25% and 19.8% for patients with LNR >25%, respectively (*P* =.017), while the 5-year OS rate was 41.3% for patients with LNR ≤25% and 25.8% for patients with LNR >25%, respectively (*P* =.003). Multivariate analysis revealed that an increased LNR was significantly associated with a poorer DFS (HR = 2.12, 95% CI, 1.32-3.41, *P* =.002) and OS (HR = 2.29, 95% CI, 1.37-5.12, *P* =.001).^69^

In 145 patients with stage I, OCCC lymph node metastases were found in 4.8% of cases, 4.1% of which were isolated metastases. Nodal involvement was identified in 10.3% of patients with positive peritoneal cytology, compared with 2.8% of those with negative cytology. Additionally, nodal metastases were detected in 11.8% of ovarian surface involvement, compared with 2.8% of those without ovarian surface involvement. Moreover, nodal metastases increased up to 37.5% for positive cytology and ovarian surface involvement.^70^ Para-aortic nodal involvement was found in 7.3% of patients with apparent early-stage OCCC.^71^ The necessity of lymphadenectomy in early OCCC is still under debate. Table 1 shows the main studies of the surgical approach.

**Table 1.** Main studies about surgical treatment (NACT vs PDS) and lymphadenectomy.

Trial	Phase	*N* of patients	Design	Results
Vergote et al. (2000)^58^	Retrospective	338	OS in NACT vs PDS in stage III-IV ovarian cancer	Patients with stage IV disease, total metastatic tumor load greater than 1.000 g, uncountable plaque-shaped peritoneal metastases, and/or poor performance status are the best candidates for NACT
Kehoe et al. (2015) ISRCTN74802813^59^	III	550	OS in NACT vs PDS in stage III-IV ovarian cancer	Survival with NACT is noninferior to PDS in women with stage III or IV ovarian cancer
Onda et al. (2016) UMIN000000523^60^	III	301	Treatment invasiveness in NACT followed by IDS vs PDS in stage III-IV ovarian, tubal, and peritoneal cancer	NACT followed by IDS is less invasive than PDS
Takano et al. (2006)^61^	Retrospective	254	Prognostic factors in patients with advanced OCCC	Cytoreductive surgery with no residual tumor improves prognosis
Suzuki et al. (2014)^62^	Retrospective	165	Survival impact of surgical staging in stage I OCCC	Optimal surgical staging is the only independent prognostic factor for RFS
Suzuki et al. (2008)^63^	Retrospective	205	Survival impact of systemic retroperitoneal lymphadenectomy in stage pTI-IIb OCCC	There is no significant difference in OS and DFS rates
Takano et al. (2009)^64^	Retrospective	199	Survival impact of complete surgical staging in stage pT1 OCCC	Peritoneal cytology status is more important than complete surgical staging
Yamazaki et al. (2018)^65^	Retrospective	127	Therapeutic significance of full lymphadenectomy in stage pT1-2 OCCC	Patients with older age and positive peritoneal cytology may benefit from full lymphadenectomy
Magazzino et al. (2011) MITO9^66^	Retrospective	240	Prognostic factors in OCCC patients	Lymphadenectomy has a strong prognostic impact on DFS and OS. The addition of paclitaxel to platinum-based chemotherapy does not affect the outcome
Mahdi et al. (2013)^67^	Retrospective	1897	Prevalence and prognostic impact of lymph node involvement in patients with clinically stage I OCCC	Lymph node metastases are uncommon, but patients with positive nodes have higher mortality rates.
Takei et al. (2018)^68^	Retrospective	68	Impact of the number of removed lymph nodes on RFS in stage I OCCC	Number of removed lymph nodes ≥35 is an independent predictor for improved RFS
Nie et al. (2019)^69^	Retrospective	265	Prognostic significance of LNR in stage III OCCC	LNR is an independent survival predictor
Mueller et al. (2016)^70^	Retrospective	145	Rate of lymph node metastasis in clinically stage I OCCC and factors associated with nodal metastasis	The rate of nodal metastasis is 4.8%. Patients with both ovarian surface involvement and positive cytology have the highest incidence of nodal metastasis
Bogani et al. (2017)^71^	Retrospective	290	Prevalence of lymph node involvement in early-stage EOC and prognostic value of lymph node dissection	High-grade serous and bilateral early-stage EOC are at high risk of nodal metastasis in both pelvic and para-aortic regions

DFS: disease-free survival, EOC: epithelial ovarian cancer, IDS: interval debulking surgery, LNR: lymph nodes ratio, OS: overall survival, PSD: primary debulking surgery, RFS: recurrence-free survival.

## Fertility-sparing surgery

As ~12% of OCCC occur in patients of reproductive age, the role of fertility-sparing surgery (FSS) represents a significant issue. FSS includes unilateral salpingo-oophorectomy with comprehensive surgical staging, preserving the uterus and the unaffected ovary. According to recent guidelines from the National Comprehensive Cancer Network (NCCN) and the European Society for Medical Oncology (ESMO) in collaboration with the European Society of Gynaecological Oncology (ESGO), FSS can be offered for stage IA/IC1 low-grade serous, endometrioid, or mucinous OC, but it is not recommended for OCCC.^56,57^ Several studies assessed survival outcomes of FSS in OCCC patients. A 5-year OS of 100% for stage IA and 93.3% for stage IC, and a 5-year PFS of 100% for stage IA and 66.0% for stage IC were reported.^72^ Park et al. found no significant difference concerning recurrence time (19 vs 20 months, *P* =.935), recurrence site, 5-year DFS (77% vs 84%, *P* =.849), and OS (91% vs 88%, *P* =.480) between FSS and radical surgery in stage I OCCC patients.^73^

A large retrospective study reported that FSS had no impact on 5-year OS or cancer-specific survival (CSS) among 741 premenopausal women with stage IA/IC OCCC. Specifically, in patients with stage IA disease who underwent FSS, the 5-year OS and CSS rates were 91.7% and 94.4%, respectively, compared with 90.7% (*P* =.400) and 90.7% (*P* =.220) for patients who underwent debulking surgery. Similarly, in patients with stage IC disease, the 5-year OS and CSS rates were 88.7% and 88.7%, respectively, compared with 70% (*P* =.090) and 70% (*P* =.100), respectively, in the standard debulking surgery group.^74^ Another multicenter study, including 164 FIGO stage I OCCC women aged <45 years revealed no statistically significant difference in OS between those who underwent FSS and those who received radical surgery (*P* =.1593). However, the status of the ovarian capsule was identified as an independent prognostic factor for OS (stage IC2/IC3 vs stage IA/IC1: HR = 4.293; 95% CI, 1.140-16.422, *P* =.0318) in a multivariable analysis.^75^ Similar results were obtained by other authors.^76^ Obstetrical outcomes should be also analyzed, and 4 deliveries with healthy neonates without pregnancy complications were reported.^76^

In a retrospective series of 8 OCCC patients who underwent FSS, none experienced recurrence during a median follow-up period of 95.4 months. Additionally, 5 patients achieved successful pregnancies.^49^

FSS appears to be a viable option for stage IA and IC1 OCCC patients who desire to preserve their fertility (Table 2).

**Table 2.** Main studies about FSS in OCCC.

Trial	Phase	*N* of patients	Design	Results
Satoh et al. (2010)^72^	Retrospective	211	Clinical outcomes and fertility in patients treated conservatively for unilateral stage I EOC	FSS is a safe treatment for stage IA patients with favorable histology, including stage IA OCCC patients
Park et al. (2015)^73^	Retrospective	47	Outcomes of FSS among young women with stage I OCCC	FSS is a safe alternative for young women with stage I OCCC who wish to preserve fertility
Nasioudis et al. (2017)^74^	Retrospective	741	Oncologic impact of uterine and ovarian preservation in premenopausal women with stage IA or IC OCCC	Uterine and ovarian preservation do not have a negative impact on oncologic outcomes
Kajiyama et al. (2019)^75^	Retrospective	164	Long-term oncologic outcomes in young patients with OCCC	OCCC patients staged greater than IC2/IC3 have an increased risk of mortality
Yoshihara et al. (2019)^76^	Retrospective	103	Clinical characteristics, prognostic factors, and effects of FSS in young patients with stage I OCCC	FSS is safe in stage IA1 and IC1 OCCC patients who wish to preserve fertility
Manning-Geist et al. (2022)^49^	Retrospective	182	Clinicopathologic and treatment factors associated with oncologic outcomes in patients with early-stage OCCC undergoing complete staging and in a subset of patients undergoing FSS	Patients with stage IA/IC1 disease have an excellent prognosis, regardless of chemotherapy. Aberrant p53 expression may portend worse outcomes. FSS seemed to be safe in patients with stage IA/IC1 disease

EOC: epithelial ovarian cancer, FSS: fertility-sparing surgery, OCCC: ovarian clear cell carcinoma.

## Role of chemotherapy

The standard systemic treatment for OCCC has been established by trials that included mainly HGSC. However, OCCC is characterized by low chemosensitivity.^61,77^ The NCCN guidelines recommend administering 6 cycles of either single-agent carboplatin or the combination of paclitaxel + carboplatin as adjuvant chemotherapy for all FIGO stages, except for patients with FIGO stage IA. In contrast, the ESMO/ESGO guidelines suggest that adjuvant chemotherapy could be omitted for FIGO stages IA-IC1 OCCC patients who undergoing complete surgical staging.^56,57^

Adjuvant chemotherapy in early-stage OCCC after surgical staging is still debated, with controversial data based on heterogeneous retrospective analyses.

A combined analysis of 2 randomized clinical trials in early-stage EOC examined 925 patients treated for early-stage EOC, including 130 OCCC. An improved 5-year OS was found in patients with early-stage OCCCs who received adjuvant chemotherapy compared with those who underwent observation in both univariate (82% vs 74%; *P* =.008) and multivariate analyses (89.2% vs 86.2%; *P* <.001).^77^

However, a retrospective analysis did not confirm significant benefit in PFS and OS for adjuvant chemotherapy in FIGO stage I OCCC patients (5-year OS rates: 85% vs 83%; *P* =.434). This lack of benefit persisted even when the analyses were stratified by stage (FIGO stage IA/IB 5-year OS rates: 87% vs 84%; *P* =.620; FIGO stage IC 5-year OS: 83% vs 80%; *P* =.620).^78^ These results in stage IA and IC1 OCCC patients were independently confirmed by other authors.^79-81^

A review and a systematic review and meta-analysis confirmed that adjuvant chemotherapy did not improve 5-year DFS and 5-year OS rates for stage IA and IB OCCC patients. However, for stage IC2/3, OCCC patients who received adjuvant chemotherapy an improvement in 5-year OS (OR = 4.98, 95% CI, 1.12-22.22; *P* =.04) and 5-year DFS (OR = 3.23; 95% CI, 0.79-13.16; *P* =.10) were reported.^82,83^

A phase III randomized controlled trial is currently ongoing and may provide more evidence on the role of adjuvant chemotherapy in FIGO stage I OCCC.

Regarding chemotherapy duration, the GOG157 trial compared 3 vs 6 cycles of adjuvant carboplatin + paclitaxel in patients with high-risk, early-stage EOC, indicating that 3 additional cycles of chemotherapy increased toxicity without significantly reducing the risk of recurrence.^84^ Several studies confirmed that early-stage OCCC patients had comparable recurrence and survival outcomes when treated with either 3 or 6 cycles of adjuvant platinum and taxane adjuvant, with no significant advantage for 6 cycles.^37,85^

OCCC has low chemosensitivity, and the response rate (RR) to a combination of carboplatin + paclitaxel is higher than other platinum-based chemotherapy regimens, such as cisplatin/cyclophosphamide, cisplatin/cyclophosphamide/adriamycin, and carboplatin/cyclophosphamide, with RRs ranging from 22% to 56% compared with 11% to 27%.^32,61,86-88^ Numerous trials have tested various regimens and modified administration protocols, but none of them have shown a significant survival advantage.^89-92^

In a recurrence setting, when feasible, secondary cytoreduction and second-line platinum-based chemotherapy in platinum-sensitive recurrence have been established as the optimal approach,^56^ with an overall RR of 80%.^93^ Conversely, platinum-resistant patients should receive mono-chemotherapy in a palliative setting, as their prognosis is dismal.^94,95^Table 3 shows that the main studies performed on systemic therapies.

**Table 3.** Main practice-changing trials in the systemic therapy.

Trial	Phase	*N* of patients	Design	Results
ICON1 + ACTION^77^	III	925	Platinum-based adjuvant chemotherapy with observation following surgery	OS at 5 years of 82% vs 74% in the observation arm
Adjuvant chemotherapy in patients with stage I endometrioid or clear cell ovarian cancer in the platinum era^78^	Cohort study	1995	platinum-based adjuvant chemotherapy with observation following surgery in a retrospective study	5-year OS rate 85% vs 83% (*P* = .439)
GOG157^84^	III	130	3 vs 6 cycles of adjuvant chemotherapy	No difference in DFS
Bevacizumab in First-Line Chemotherapy Improves Progression-Free Survival for Advanced Ovarian Clear Cell Carcinoma	Retrospective	28	First-line CT vs CT + Bevacizumab	mPFS of 12.0 vs 29.8
Low response rate of second-line chemotherapy for recurrent or refractory clear cell carcinoma of the ovary: a retrospective Japan Clear Cell Carcinoma Study^94^	Retrospective	75	Retrospective evaluation of chemotherapy response rates in recurrent OCCC	ORR 6% in platinum-resistant disease
Lack of effective systemic therapy for recurrent clear cell carcinoma of the ovary^95^	Retrospective	51	Retrospective evaluation of chemotherapy in recurrent OCCC	ORR 1% in platinum-resistant disease

CT: chemotherapy, DFS: disease-free survival, OCCC: ovarian clear cell carcinoma, ORR: objective response rate, OS: overall survival, PFS: progression-free survival.

## Targeted therapies

The most common gene alterations in OCCC are ARID1A and PIK3CA mutations, which have been detected in 15%-62% and 31.5%-55% of the cases, respectively.^96-98^ The ARID1A mutation results in the loss of expression of the BAF250a protein. PIK3CA mutations tend to occur in ARID1A-deficient tumors, and they cooperate in carcinogenesis by deregulating the production of IL-6.^99-101^

**Figure 1.**
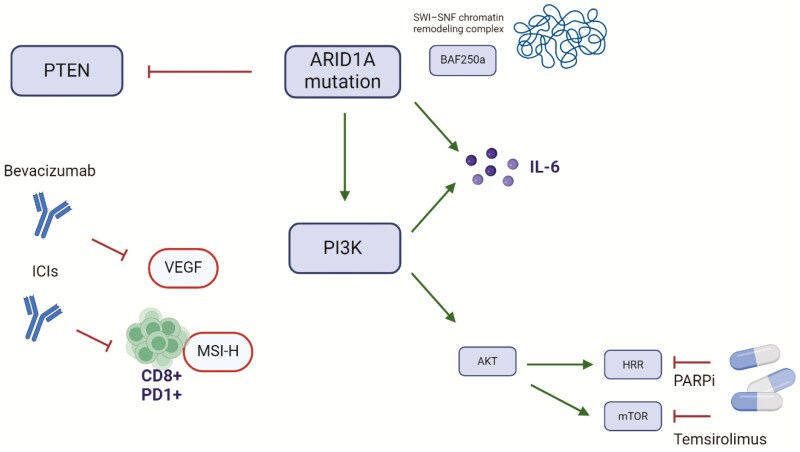
Main molecular alterations and possible therapeutic targets for OCCC. ARID1A mutation activates the PI3K/ATK/mTOR pathway that interacts with the homologous recombination repair (HRR) system and leads to an increased production of IL-6. ARID1A mutation is also involved in the inhibition of PTEN and the loss of expression of the BAF250a protein. PARPi and Temsirolimus can target, respectively, HRR system and mTOR, while bevacizumab and ICIs interact with VEGF and tumors that present microsatellite instability.

OCCC frequently shows overexpression of Hypoxia-Induced transcription Factor (HIF1α) and PTEN mutations, according to the hypothesis of an aberrant PI3K/PTEN signaling pathway in carcinogenesis.^12,102-105^ Less common alterations in OCCC include KRAS mutations (5%-10%) and AKT2 amplification (14%).^12,103^ MET overexpression, gene amplification, or MMRd has been detected in 22%-37% of cases, while somatic or germline BRCA1-2 mutations have been identified in 6% of cases. Moreover, p53 profile is typically wild-type in OCCC, with a frequency of mutations of ~8.5%-21.6%.^12,21,106-108^Figure 1 summarizes the main molecular alterations and possible therapeutic targets.

## Antiangiogenic therapy

Bevacizumab can be considered in both first-line and relapsed disease. However, data regarding its benefits were lacking and the approval was based on the large phase III trials which included few OCCC. Furthermore, bevacizumab did not demonstrate significant activity in OCCC.^109,110^

A multicenter, retrospective analysis reported a benefit in both PFS (12.5 vs 29.7 months; *P* =.006) and OS (34.7 vs 51.4 months; *P* =.027) in patients treated with bevacizumab in addition to adjuvant chemotherapy for advanced OCCC. The benefit was more pronounced for FIGO Stage IIIA/B (HR: 0.35; 95% CI, 0.15-0.78) and for patients with less than 300cc of ascites (HR = 0.46; 95% CI, 0.36-1.06).^111^

## PARP inhibitors

Maintenance therapy with PARP inhibitors (PARPi) is indicated for endometrioid and HGSC subtypes. OCCC patients with BRCA mutations may benefit from PARPi, but no trials have tested this possibility.

In a preclinical model, a correlation between ARID1A expression levels, PI3K/Akt1 pathway activity, and the efficacy of a DNA homologous recombination repair (HRR) system was observed. ARID1A-mutated OC cells exhibited significant sensitivity to PARPi, in contrast to wild-type cells.^112^ In another study, OCCC cell lines were treated with PARPi, including AG14361, Veliparib, or Olaparib either alone or in combination with other cytotoxic drugs for 72 hours. OCCC demonstrated heightened susceptibility to chemo-sensitization when exposed to PARPi in combination with paclitaxel, 5-fluorouracil, carboplatin, doxorubicin, and/or cisplatin.^113^ Thus, given the close interaction between ARID1A and the homologous HRR system, it can be hypothesized that OCCC could be sensitive to PARPi treatment also in vivo.

## Immune-checkpoint inhibitors

Approximately 15% of OCCC have been found to have microsatellite instability (MSI-H). Within this subset of cases, there is a significant increase in the presence of CD8 + tumor-infiltrating lymphocytes (TILs), a higher CD8+/CD4 + ratio, and a greater presence of PD-1 + TILs when compared with microsatellite stable tumors and HGSC.^114^ The highly immunogenic environment suggests that immunotherapy, specifically immune-checkpoint inhibitors (ICI), might be an option to explore. Although trials involving the use of ICI in EOC have yielded predominantly disappointing results, with RRs ranging between 8% and 13% for single-agent therapy, subset analyses indicate that patients with OCCC may benefit more.^115-117^

Among 16 OCCC patients who received immunotherapy, 4 (25%), experienced durable clinical benefit defined as a time to treatment failure ≥180 days.^118^ A randomized phase II trial enrolled patients with recurrent OCCC who were randomized to receive Durvalumab or chemotherapy. An objective RR (ORR) of 10.7% for Durvalumab and an ORR of 18.8% for chemotherapy were achieved (*P* =.884). When patients who had previously progressed on chemotherapy and were then allowed to cross over to Durvalumab were considered, the ORR of Durvalumab increased to 13.4%. This study did not identify significant differences in ORR, PFS, or clinical benefit rate (CBR).^119^

Pembrolizumab was studied in a phase II trial and showed after a median follow-up of 2.1 years, a median PFS of 12.2 weeks (95% CI, 5.9-23.9), and a 1-year PFS rate of 22.0% (95% CI, 11.5%-34.7%) in heavily pretreated patients, suggesting a possible activity.^120^

In the NRG trial, which assessed the effectiveness of ipilimumab in combination with nivolumab compared with nivolumab alone in women with persistent or recurrent EOC, OCCC exhibited 5-fold higher odds of response compared with other subtypes.^121^

Recently, preliminary results from a phase II single-arm study that enrolled pretreated patients to receive a combination of sintilimab (anti PD-L1) and bevacizumab showed an ORR of 40%.^122^

Other ongoing trials include BrUOG 354, a phase II randomized trial evaluating nivolumab with or without ipilimumab for ovarian and extra-renal clear cell carcinomas. Stage I OCCC showed a median PFS of 2.7 months (95% CI, 1.3-5.1) for the Nivolumab-alone group and 5.1 months (95% CI, 0.9-NR) for the combination therapy group. Based on these findings, the combination arm received approval to advance to stage II of the trial, while the arm containing only Nivolumab did not show significant differences compared with chemotherapy in the same setting.^123^

## PI3K/AKT/mTOR inhibitors

The GOG-268 trial, which randomized patients to receive carboplatin + paclitaxel with or without the mTOR inhibitor Temsirolimus as adjuvant treatment for early-stage OCCC, did not demonstrate a significantly higher PFS compared with carboplatin + paclitaxel alone. However, a Japanese phase II single-arm trial investigated the combination of trabectedin and temsirolimus in recurrent OCCC and achieved an ORR of 14.3% and a CBR of 42.9%.^124,125^ This combination stems from the enhanced activity of trabectedin on nucleotide excision repair proficient cells, which are more prevalent in OCCC and may be related to platinum resistance.^103^

## Other agents

Among multiple tyrosine kinase inhibitors (mTKIs), Sunitinib showed an RR of 6.7% in the GOG-254 trial, which randomized patients with persistent or recurrent OCCC. On the other hand, Nintedanib did not show significant activity as a single agent in patients with recurrent ovarian or endometrial clear cell carcinoma in the ENGOT-OV36 trial.^126^ In another trial, patients with recurrent OCCC were randomized to receive another mTKI, ENMD-2076 (selective inhibitor of mitotic kinase Aurora A), in combination with a VEGF inhibitor. Although the trial did not demonstrate a benefit in the overall population, the loss of ARID1A correlated with improved PFS, thus suggesting that loss of ARID1A could potentially serve as a predictive biomarker for identifying patients who may benefit from this treatment.^127^ Despite the presence of MET amplification in OCCC, the use of single agent Cabozantinib, targeting MET, VEGFR2, and RET produced disappointing results in a phase II single-arm study, with no objective response among patients with recurrent OCCC enrolled.^128^

Relapsed OCCC needs alternative treatments to ineffective chemotherapies. ICI monotherapy has poor results, despite preclinical evidence. PARPi may be helpful, but platinum resistance is still a challenge. Table 4 summarizes the main ongoing trials on OCCC.

**Table 4.** Ongoing trials enrolling OCCC patients.

Trial	Phase	Therapy	Setting	Primary objectives	Status
NCT05759312	I/II	Zimberelimab combined with metformin	Relapsed/persistent OCCC	ORR	Not yet recruiting
NCT06065462	I/II	Dostarlimab and LB-100	Recurrent OCCC, peritoneal, or fallopian tube cancer	Toxicity	Recruiting
NCT05226507	I	NXP800	Recurrent ARID1a mutated, clear cell ovarian carcinoma	Toxicity, ORR	Recruiting
NCT05950464	Ib	M1774 and ZEN-3694	Recurrent ovarian and endometrial cancer	MTD and DLTs	Recruiting
NCT05498597	I	AMT-151	Advanced solid tumors	MTD	Recruiting
NCT05920798	I/II	folate receptor alpha dendritic cells (FRaDCs) plus pembrolizumab	Advanced ovarian cancer	Toxicity, ORR	Recruiting
NCT04092270	I	peposertib (M3814) in combination with pegylated liposomal doxorubicin hydrochloride (PLD)	Recurrent or persistent epithelial ovarian cancer	Safety and tolerability	Recruiting
NCT05001282	I/II	ELU001	Recurrent ovarian cancer overexpressing folate receptor alpha (FRα) overexpressing	MTD, ORR	Recruiting
NCT02142803	I	(mTOR) (TORC1/2) inhibitor MLN0128 when given in combination with bevacizumab	Advanced solid tumors which are sensitibe to bevacizumab	MTD, ORR	Not recruiting
NCT06023862 (DOVE)	II	Dostarlimab alone or with bevacizumab vs nonplatinum chemotherapy	Recurrent gynecological clear cell carcinoma	PFS	Not yet recruiting
NCT05231122	II	Pembrolizumab, bevacizumab with or without agonist anti-CD40 CDX-1140	Recurrent epithelial ovarian cancer	ORR, toxicity	Not yet recruiting
NCT04919629	II	APL-2 and pembrolizumab vs APL-2, pembrolizumab and bevacizumab vs bevacizumab alone	Recurrent epithelial ovarian cancer	Accumulation of effusion	Not yet recruiting
NCT04735861	II	Sintilimab + Bevacizumab	Recurrent OCCC	ORR	Recruiting
NCT05296512	II	Pembrolizumab lenvatinib	Recurrent or persistent OCCC	ORR, 6 months PFS	Recruiting
NCT05043922	II	CYH33	Recurrent or persistent OCCC	ORR	Recruiting

DFS: disease-free survival, DLTs: the dose-limiting toxicities, MTD: maximum tolerated dose, FOCCC: ovarian clear cell carcinoma, ORR: objective response rate.

Table 4 shows ongoing clinical trials considering patients with OCCC.

## Radiotherapy

A retrospective analysis involving 241 patients with stage IA-II OCCC found that abdominopelvic irradiation, followed by 3 cycles of adjuvant carboplatin + paclitaxel, improved DFS in stage IC2/IC3/II.^129^ In contrast, another study did not find an association between radiotherapy and survival benefits in 163 patients with stage I-II OCCC.^130^ A cohort study assessed the utility of first-line adjuvant intensity-modulated whole abdominal radiation therapy in 5 OCCC overexpressing HNF1β, showing that at a median follow-up of 77 months, all patients remained progression-free.^131^ Because of the lack of consistent evidence supporting its benefit, adjuvant radiotherapy is not part of the standard therapy for OCCC. However, in the recurrent setting, radiotherapy is used as a loco-regional treatment. A retrospective study of 55 patients, 23 of whom received radiotherapy, suggested a survival benefit for these latter patients.^132^

## Conclusions

**Figure 2.**
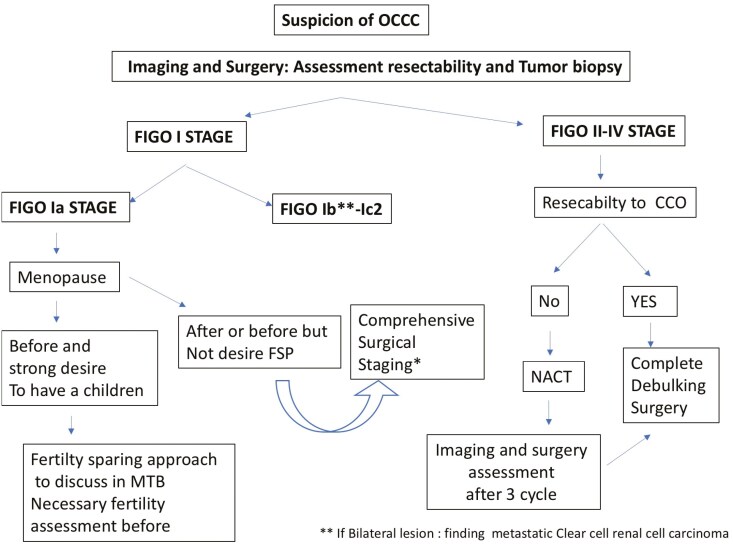
Proposal of a management algorithm for a newly diagnosed OCCC. FSS: fertility-sparing surgery, MTB: multidisciplinary tumor board, NACT: neoadjuvant chemotherapy, OCCC: ovarian clear cell carcinoma, PDS: primary debulking surgery.

**Figure 3.**
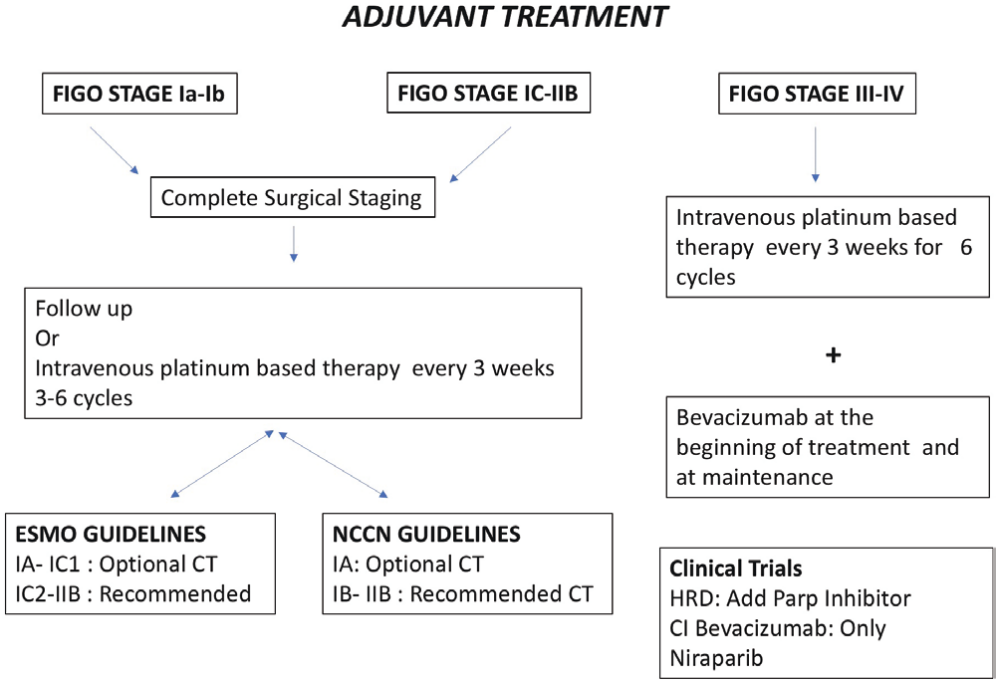
Proposal of a management algorithm for adjuvant treatment. CT: chemotherapy.

OCCC is a rare and aggressive disease with a poor prognosis in the advanced stage. Current treatments in first-line and recurrence settings come from trials where the most common histotype was HGSC, leaving many questions unanswered. However, based on the available literature, we propose an algorithm for surgical management (Figure 2) and systemic adjuvant treatment (Figure 3).

Primary surgery or IDS remains the milestone of the treatment of OCCC, and the role of LND should be confirmed for early stages. FSS approach seems to be a safe option in FIGO stage I OCCC patients, but further studies are needed to confirm these data.

The role of chemotherapy in early-stage disease is still unclear, as demonstrated by the different positions by ESMO/ESGO and NCNN guidelines: while the first suggests that chemotherapy should only be offered from stage IC, the latter indicates that the treatment can be offered in all early stages.

The role of target therapies for OCCC is worthy of further investigation. While immunotherapy has already been explored, giving promising preliminary results, PARPi has shown a strong preclinical rationale, with their close interaction between ARID1A and the HRR system. It should be investigated in a clinical setting. Similarly, combination therapies such as double ICI or antiangiogenic agent + PARPi have yet to be explored. The molecular-driven therapy approach is under investigation in the groundbreaking phase II BOUQUET trial (NCT04931342).^133^ A better understanding of the molecular landscape in OCCC might allow us to improve the poor prognosis in advanced stages.

## Data Availability

No new data were generated or analyzed in support of this research.
